# Biphasic synovial sarcoma with a striking morphological divergence from the main mass to lymph node metastasis: A case report

**DOI:** 10.1097/MD.0000000000028481

**Published:** 2022-01-07

**Authors:** Ha Young Woo

**Affiliations:** a Department of Pathology, National Cancer Center, Goyang, Republic of Korea; b Department of Pathology, Kyung Hee University Hospital, Kyung Hee University College of Medicine, Seoul, Republic of Korea.

**Keywords:** case report, lymph node metastasis, SS18-SSX translocation, synovial sarcoma, transducin-like enhancer of split 1

## Abstract

**Rationale:**

Synovial sarcoma accounts for 5% to 10% of all soft tissue sarcomas and involves almost any anatomic site, particularly the deep soft tissue of the extremities of young adults. The incidence rate of lymph node metastases in synovial sarcoma is 3% to 7%, but the detailed morphological features of the metastatic tumors in the lymph node have not been documented.

**Patient concerns:**

A 64-year-old Korean man presented with a huge mass in the left lower thorax and multiple hypermetabolic lymph nodes along the mediastinal, supraclavicular, internal mammary, and retrocrural regions.

**Diagnoses:**

The patient was diagnosed with primary pleuropulmonary biphasic synovial sarcoma with lymph node metastases, where the main mass mostly comprised spindle cells (>95%) and the metastatic lymph nodes comprised only epithelial cells.

**Interventions:**

Left lower lobe lobectomy with the resection of the chest wall (including left ribs 8-10) and diaphragm and mediastinal lymph node dissection were performed.

**Outcomes:**

In the 2-month follow-up period, there have been no complications so far, and the attending physician is currently planning for the adjuvant chemotherapy.

**Lessons:**

The main mass and the metastatic lesion can be clearly different morphologically. In tumors with biphasic differentiation, such as synovial sarcoma, cells that constitute only a small fraction of the main mass may appear as the dominant cells in metastatic lesions.

## 1 Introduction

Synovial sarcoma accounts for 5% to 10% of all soft tissue sarcomas and involves almost any anatomic site, particularly the deep soft tissue of the extremities of young adults.^[1]^ Synovial sarcoma's cell of origin has not yet been elucidated, and neural, myogenic, or multipotent mesenchymal stem cells have been reported as putative causative cells.^[2–5]^ Synovial sarcoma was initially recognized as a biphasic tumor with both epithelial and uniform spindle cell components, but it is now clear that there is evident morphological and immunohistochemical heterogeneities within this group of neoplasms. Morphologically, synovial sarcoma displays a monotonous spindle cell component, which is typically arranged in long fascicles or herringbone pattern, with varying degrees of epithelial differentiation.^[1]^ Despite its histological diversity, synovial sarcoma is molecularly defined by a single alteration: the t(X;18)(p11.2;q11.2) translocation, involving the SS18 (formerly SYT) gene on chromosome 18 and 1 of several synovial sarcoma X (SSX) genes on chromosome X (usually SSX1 or SSX2).

Synovial sarcoma typically presents in the extremities, particularly the proximal or middle portions of the lower extremities (most commonly around the knee) and especially in periarticular regions, hence the historical misnomer “synovial sarcoma”. Besides the common anatomic sites, synovial sarcoma has also been also reported at almost every anatomic site. After the extremities, the trunk is the second most frequent site, followed by the head and neck regions.^[6]^ Synovial sarcoma can occur in the thorax, including the anterior or posterior mediastinum, pleuropulmonary region, chest wall, lumbar spine, and heart,^[7]^ representing approximately 1% to 5% of cases.^[6]^

Herein, we reported an unusual case of molecularly confirmed pleuropulmonary synovial sarcoma with clearly distinct morphological features in the main mass and lymph nodes.

## 2 Case presentation

**Figure 1:**
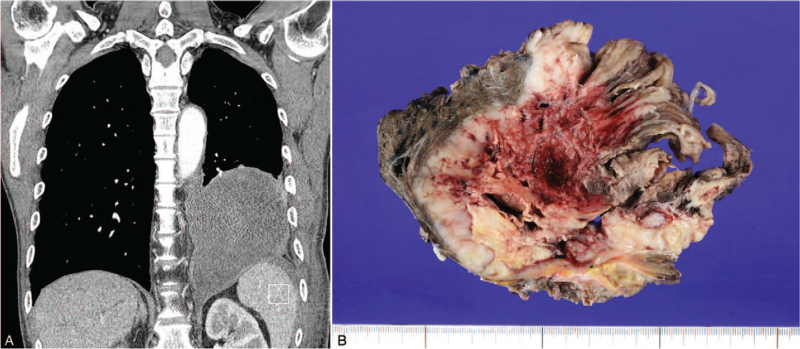
(A) Coronal computed tomography image demonstrates a well-defined mass with internal necrosis in the left lower hemithorax. (B) Gross specimen shows a well-defined solid mass, which contains geographic necrosis and hemorrhage and involves the lung (the left side of the photo), ribs (the bottom of the photo), and diaphragm (the right side of the photo).

A 64-year-old Korean man presented with cough and sputum for 2 weeks. He was a smoker and had no significant past medical or surgical history. A chest radiograph revealed a huge intrathoracic mass associated with pleural effusion in the left side. A contrast-enhanced computed tomography (CT) of the chest revealed an approximately 13.0-cm-sized well-defined mass with internal necrosis, which was located in the left mid-to-lower hemithorax (Fig. 1A). Radiologically, it was difficult to define exactly whether the tumor was of pleural or pulmonary origin, as the mass was attached to both the left pleura and left lung. In positron emission tomography (PET)-CT, a hypermetabolic mass was noted in the left thoracic region. Additionally, multiple hypermetabolic lymph nodes were found in the left internal mammary, left supraclavicular, and right retrocrural region, suggesting the possibility of metastasis. A diagnostic video-assisted thoracic surgery with biopsy was performed. Microscopically, the tumor showed the proliferation of monotonous spindle cells with immunohistochemical expression of epithelial membrane antigen, B-cell leukemia/lymphoma 2, and transducin-like enhancer of split 1 (TLE1). The pathological differential diagnoses for biopsy included monophasic synovial sarcoma, sarcomatoid carcinoma, sarcomatoid mesothelioma, and malignant solitary fibrous tumor.

A surgery was scheduled, and left lower lobe lobectomy with the resection of the chest wall (including ribs 8-10) and diaphragm and mediastinal lymphadenectomy were performed. During the operation, multiple pleural seeding nodules were noted. The surgical specimens were submitted to the department of pathology. On gross examination, a 13.7-cm-sized well-defined whitish mass, involving the lung parenchyma, chest wall, and diaphragm, was noted (Fig. 1B). The mass had internal geographic necrosis and hemorrhage.

**Figure 2:**
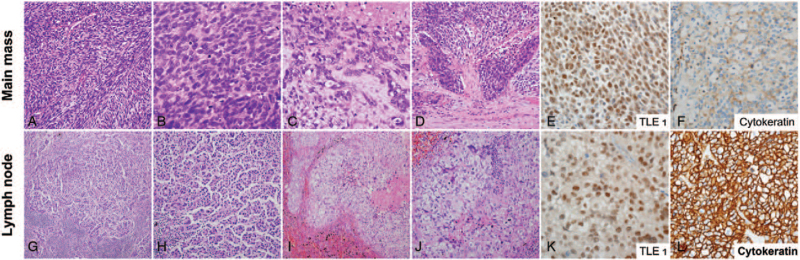
The representative images of the main mass (A-F) and the metastatic lymph node (G-L). The main mass predominantly (>95%) comprised dense cellular sheets and fascicles of uniform spindle cells (A) with ovoid vesicular nuclei, inconspicuous nucleoli, and sparse eosinophilic cytoplasm (B). At the periphery of the tumor, a small fraction (<5%) of the tumor exhibited epithelial differentiation, forming varying-sized cords (C) and solid nests (D). The tumor cells were diffusely and strongly immunostained with transducin-like enhancer of split 1(TLE1) (E) and focally and weakly with pan-cytokeratin (F). The metastatic lymph nodes showed purely epithelial component in micropapillary architecture (G and H, frozen section) and solid nests (I and J). The tumor cells were diffusely and strongly immunostained with TLE1 (K) and pan-cytokeratin (L). (Original magnification: [A] ×200, [B and C] ×400, [D] ×200; [E-F] ×400, [G] ×100, [H] ×200, [I] ×100, [J] ×200, [K and L] ×400).

Microscopically, the vast majority (>95%) of the tumor comprised monotonous spindle cell proliferation in long fascicular growth or herringbone pattern, with a minor proportion (<5%) of epithelial component. The spindle cell component comprised dense cellular sheets and fascicles of uniform spindle cells with ovoid vesicular nuclei, inconspicuous nucleoli, and sparse eosinophilic cytoplasm (Fig. 2A-B). The epithelial component was observed only in the periphery of the mass, and the amount was significantly minimal that it could easily be overlooked without careful examination. The epithelial component formed varying-sized solid nests and cords, in which there were occasional small glandular lumina with scattered intercellular slits (Fig. 2C-D). There were no overt histologic features of well-formed tubular or papillary architecture. The epithelial cells were polygonal or cuboidal and had ovoid vesicular nuclei with inconspicuous nucleoli and abundant palely eosinophilic cytoplasm. Focal areas of gradual transition between the 2 components were present. The tumor contained areas of coagulative necrosis and increased mitotic activity (35 mitoses per 10 high-power fields). A number of lymphatic invasions were observed at the border between the tumor and normal lung parenchyma.

The mediastinal and peribronchial lymph nodes were submitted and revealed metastases in 3 lymph nodes (2 left lower paratracheal and 1 ipsilateral peribronchial). Interestingly, the metastatic tumor cells in the lymph nodes demonstrated overt epithelial cell proliferation in micropapillary pattern and solid nests, strikingly mimicking a metastatic carcinoma (Fig. 2G-L). The lymph nodes were first received for frozen section examination, and the pathologist established a frozen diagnosis of “metastatic carcinoma” (Fig. 2G-H), because the previous biopsy specimen consisted entirely of monotonous spindle cells. In the permanent specimen, the metastatic tumor cells in the lymph nodes were morphologically relatively different from those of the main mass. The epithelial component of the main mass appeared less differentiated, only forming solid nests with occasional scattered intercellular slits and cords, whereas the tumor cells in the lymph nodes were composed of entirely well-formed micropapillary structures and varying-sized solid nests. As the morphological discrepancy between the main mass and lymph nodes was observed, we needed to rule out the possibility of true metastatic carcinoma of unknown origin. To ensure that there was no other hidden malignancy in the lung, we carefully inspected and vigorously sampled the lung. As a result, there were no additional abnormal lesions in the lung parenchyma. The other lymph nodes that were hypermetabolic on PET-CT were not surgically resected; thus, it was not known whether the tumor metastasized to the lymph nodes.

**Figure 3:**
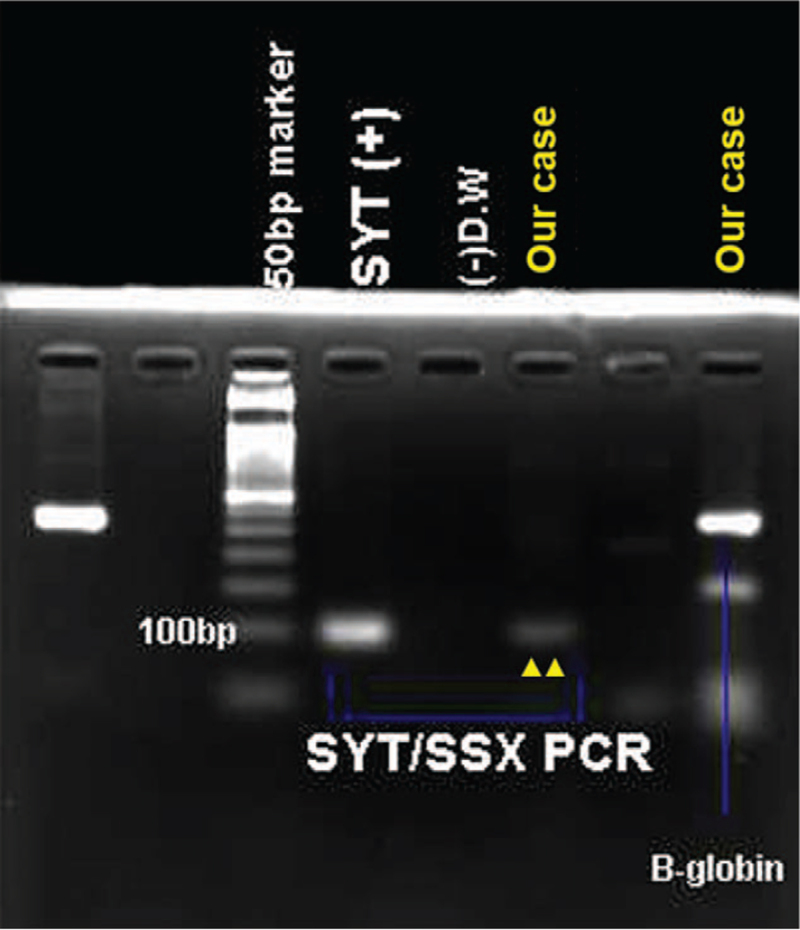
The result of real-time polymerase chain reaction for SS18 (SYT)-SSX translocation confirms the presence of the translocation in our case (yellow arrow).

Immunohistochemically, spindle cell and epithelial cell components showed diffusely and strongly nuclear staining with TLE1, and some areas of both components displayed focally weakly cytoplasmic staining for pan-cytokeratin (Fig. 2E,F,K,L). To rule out the possibility of lung primary adenocarcinoma, TTF1 immunostaining was performed, and it was negative in both the main mass and lymph node. Finally, real-time polymerase chain reaction confirmed the presence of SS18 (SYT)-SSX translocation (Fig. 3). Taken together, we concluded that the main mass of biphasic synovial sarcoma predominantly consisted of spindle cells, but metastasis to lymph nodes showed overt epithelial cells, showing more pronounced epithelial differentiation.

During the 2-month follow-up period, there have been no complications so far, and the attending physician is currently planning for the adjuvant chemotherapy.

## 3 Discussion

Synovial sarcoma is a malignant mesenchymal neoplasm of uncertain origin. Morphologically, synovial sarcomas can display monophasic or biphasic morphology. Monophasic types usually consist exclusively of monomorphic spindle cells, with exceptionally rare cases comprising epithelial components.^[8]^ Biphasic tumors comprise variable proportions of monomorphic spindle cells and epithelial cells, which can form glands or solid nests and cords.^[9]^ Poorly differentiated synovial sarcomas demonstrate in part or entirely of a highly cellular proliferation of primitive round to spindle cells with frequent rhabdoid morphology, brisk mitotic activity, and necrosis.^[9–12]^

Soft tissue sarcomas typically spread via the hematogenous route,^[13]^ but approximately 2% to 10% of patients with sarcoma develop lymph node metastases.^[14,15]^ Nevertheless, a significantly higher risk for lymph node metastases has been reported for certain subtypes, including synovial sarcoma, clear cell sarcoma, epithelioid sarcoma, and rhabdomyosarcoma.^[15–18]^ Historically, the frequency of lymph node metastasis in synovial sarcoma ranges from 14% to 50%^[15,17,19]^; thus, the need for sentinel lymph node biopsy in synovial sarcoma has been discussed.^[20,21]^ Certainly, prior studies over the past few decades have mostly dealt with cases diagnosed without molecular confirmation. More recent data have reported that the risk of lymph node metastasis of synovial sarcoma is approximately 3% to 7%, lower than previously considered.^[1,22–24]^ Keung et al^[1]^ reported that the intrathoracic synovial sarcomas showed the highest rate of lymph node metastases (6.6%) among the various anatomic sites, including the trunk/extremity (2.5%), head/neck/face (5.6%), and intra-abdominal/visceral (4.1%) location.

In other malignancies, in particular hepatocellular carcinoma, even if only a small fraction of the hepatocellular carcinoma cells (reportedly >5%) express stemness-related markers (keratin [K]19), they tend to spread more often to the lymph nodes and behave aggressively.^[25,26]^ The stemness-related marker, such as K19, is also a marker for the hepatic progenitor cells, which have capacity to differentiate into both hepatocytes and cholangiocytes. Although the exact cause is yet known, K19 is usually expressed in cells exhibiting cholangiocellular differentiation at the periphery of the tumor mass. When the cells expressing K19 show clear adenocarcinomatous morphology, it can be classified as combined hepatocellular cholangiocarcinoma. This finding is similar to our case in that cells showing distinct differentiation were observed in the periphery of the tumor and the cells metastasized to the lymph nodes. These morphological similarities found in different tumor types may help to understand tumor biology, especially the biphenotypic tumors, such as synovial sarcoma and combined hepatocellular cholangiocarcinoma.

To the best of our knowledge, in previous reports of synovial sarcoma with lymph node metastasis, there were no specific descriptions on which cells the tumors that have metastasized to the lymph nodes are consisted of. In our case, the main mass consisted of overwhelming spindle cell component (>95%) with a minimal fraction of epithelial cell component (<5%), consistent with biphasic synovial sarcoma with confirmed translocation. However, interestingly, the lymph node metastasis lesion displayed pure epithelial cell proliferation, strikingly mimicking a metastatic carcinoma. Certainly, the frozen section for the metastatic lymph node was interpreted as “metastatic carcinoma”. A whole-body PET-CT and chest CT revealed no other abnormal findings suggestive of other primary foci. In addition, a repetitive and meticulous gross examination was conducted to confirm the absence of other lung carcinomas in the submitted specimen, but there were no abnormal findings. The metastatic lymph node showed diffuse and strong nuclear expression by TLE1 immunostaining, confirming the metastatic synovial sarcoma to the lymph node. Taken together, we present an unusual case of the primary pleuropulmonary biphasic synovial sarcoma with distinct morphological divergence between the main mass and the nodal metastatic lesion.

## 4 Conclusion

We reported an unusual case of the primary pleuropulmonary biphasic synovial sarcoma with distinct morphological divergence between the main mass and the nodal metastatic lesion. Moreover, our findings are of significant relevance to clinicians who manage patients with synovial sarcoma. I believe that clinicians and pathologists would significantly benefit by reading this article.

## Acknowledgments

I thank the Dr. SY.P. who diagnosed this case and interpreted the real-time polymerase chain reaction result.

## Author contributions

HYW reviewed the case, provided clinical data and follow-up information, wrote the manuscript, and submitted the final version of the manuscript.

**Conceptualization:** Ha Young Woo.

**Data curation:** Ha Young Woo.

**Formal analysis:** Ha Young Woo.

**Investigation:** Ha Young Woo.

**Methodology:** Ha Young Woo.

**Writing – original draft:** Ha Young Woo.

**Writing – review & editing:** Ha Young Woo.
